# Scientific Publication Patterns of Mobile Technologies and Apps for Posttraumatic Stress Disorder Treatment: Bibliometric Co-Word Analysis

**DOI:** 10.2196/19391

**Published:** 2020-11-26

**Authors:** Atik Kulakli, Ivanna Shubina

**Affiliations:** 1 Department of Management Information Systems College of Business Administration American University of the Middle East Egaila Kuwait; 2 Psychology, General Education Liberal Arts Department American University of the Middle East Egaila Kuwait

**Keywords:** posttraumatic stress disorder (PTSD), mobile technologies, mobile apps, treatment, text analysis, co-word analysis, bibliometric, Web of Science

## Abstract

**Background:**

Mobile apps are viewed as a promising opportunity to provide support for patients who have posttraumatic stress disorder (PTSD). The development of mobile technologies and apps shows similar trends in PTSD treatment. Therefore, this emerging research field has received substantial attention. Consequently, various research settings are planned for current and further studies.

**Objective:**

The aim of this study was to explore the scientific patterns of research domains related to mobile apps and other technologies for PTSD treatment in scholarly publications, and to suggest further studies for this emerging research field.

**Methods:**

We conducted a bibliometric analysis to identify publication patterns, most important keywords, trends for topicality, and text analysis, along with construction of a word cloud for papers published in the last decade (2010 to 2019). Research questions were formulated based on the relevant literature. In particular, we concentrated on highly ranked sources. Based on the proven bibliometric approach, the data were ultimately retrieved from the Web of Science Core Collection (Clarivate Analytics).

**Results:**

A total of 64 studies were found concerning the research domains. The vast majority of the papers were written in the English language (63/64, 98%) with the remaining article (1/64, 2%) written in French. The articles were written by 323 authors/coauthors from 11 different countries, with the United States predominating, followed by England, Canada, Italy, the Netherlands, Australia, France, Germany, Mexico, Sweden, and Vietnam. The most common publication type was peer-reviewed journal articles (48/64, 75%), followed by reviews (8/64, 13%), meeting abstracts (5/64, 8%), news items (2/64, 3%), and a proceeding (1/64, 2%). There was a mean of 6.4 papers published per year over the study period. There was a 100% increase in the number of publications published from 2016 to 2019 with a mean of 13.33 papers published per year during this latter period.

**Conclusions:**

Although the number of papers on mobile technologies for PTSD was quite low in the early period, there has been an overall increase in this research domain in recent years (2016-2019). Overall, these findings indicate that mobile health tools in combination with traditional treatment for mental disorders among veterans increase the efficiency of health interventions, including reducing PTSD symptoms, improving quality of life, conducting intervention evaluation, and monitoring of improvements. Mobile apps and technologies can be used as supportive tools in managing pain, anger, stress, and sleep disturbance. These findings therefore provide a useful overview of the publication trends on research domains that can inform further studies and highlight potential gaps in this field.

## Introduction

Mobile technologies have emerged as useful tools to support patients who have posttraumatic stress disorder (PTSD) [1]. Recent studies in this field have mainly focused on the development and evaluation of mobile health (mHealth) tools [2], assessing their efficacy within a health care field [3], and developing a standard for creating PTSD-related mHealth apps [4].

New mobile technologies are accessible and cost-effective tools to assist in identifying PTSD symptoms [5,6] and suicidal behaviors [7]. Mobile technologies are supportive in prognosticating the impact that PTSD symptoms have on treatment efficiency [8], in the evaluation of the effectiveness of psychotherapy [9,10] and its precision [11], and in helping individuals manage their mental health [12,13]. The most common benefits of using mobile interventions are cost-effectiveness, reducing waiting lists, and high accessibility [5].

Thus, mHealth tools have potential for supporting traditional treatment for mental disorders among veterans with PTSD [14]. Mood Tracker [15] and other mHealth apps (eg, Parenting 2GO, PTSD Coach, and PTSD Family Coach) were established for the treatment of military staff diagnosed with PTSD and their families [16], which have been demonstrated to be feasible and relevant. In addition, the efficiency of a mobile app designed to screen trauma-related symptoms as a diagnostic tool for trauma survivors was proven [8].

New strategies for a self-managed and web-based wellness training program for PTSD veterans are recognized as being accessible, low-cost, and efficient to decrease PTSD symptoms [5]. A similar study showed no significant difference in effectiveness between a mindfulness training program delivered by a psychotherapist and the same program provided through a self-directed mobile app [6]. Fraynt et al [17] explored the ability of a mobile app to help support the transition to civilian life among PTSD veterans. Similarly, Pavliscsak et al [18] demonstrated the importance of engagement with an mHealth app among service members with PTSD symptoms in transition to help improve communication skills.

In addition, several studies have examined the use of mobile apps in treatment for smokers with PTSD [19,20] and their use in integrated care [12,13], demonstrating their effectiveness and congruency. A pilot study on the mobile app PTSD Coach demonstrated a significant improvement in quality of life among patients with PTSD [21]. Another study showed the efficiency of the Moodivate app in managing limitations associated with evidence-based psychotherapy by decreasing the symptoms of PTSD and depression among adults [22,23]. Other studies on mobile apps for the treatment of anger symptoms [24,25] and in managing pain among individuals with PTSD [26,27] showed feasibility and therapeutic benefits. Overall, mHealth has been evaluated to be effective in reducing emotional dysregulation among veterans with PTSD [28]. Other comparative studies between in vivo exposure and virtual reality–based exposure therapies for patients with PTSD symptoms indicated that the virtual experience was considered to be a more flexible approach [29,30].

An influential group of studies has focused on how mobile technology can be useful in applying cognitive behavioral therapy (CBT) for various mental health conditions. The special needs of patients with PTSD create a demand for modified approaches other than therapeutic sessions in vivo [31] and more creative tools used in treatment [32]. Recent systematic reviews [9,10,30] have explored the efficacy of the mHealth apps embodying CBT principles for the treatment of various mental disorders, including PTSD. For example, Martinez-Miranda et al [7] assessed the effectiveness of applying a mobile-based app in recognizing individuals diagnosed with PTSD and demonstrating suicidal behaviors. Wang et al [33] indicated the high potential and efficiency of mobile apps in the monitoring and management of mental disorders, including PTSD. Stirman et al [34] assessed an app designed for creating and assessing universal worksheets to help evaluate the accuracy of CBT therapy sessions for patients with PTSD. Evans et al [35] investigated the development of a cognitive assessment tool via mobile technology. A similar study by Price et al [36] suggested that PTSD checklists delivered through a mobile app or on paper were equally efficient.

Therefore, new technology as a psychological tool in treating PTSD has been explored by researchers in various modalities, including mobile apps, mHealth, web-based programs, virtual reality, checklists, and assessments [30]. However, the majority of mHealth apps that are currently available lack clinically validated evidence of their efficacy [3]. Accordingly, the primary aim of this study was to explore scientific publication patterns in the research domain of “mobile technologies and apps” concerning PTSD. We further aimed to reveal the contribution of scientific knowledge by highlighting the gaps and provide new directions of potential development areas for further studies.

## Methods

### Research Questions

Based on the research scope and objectives, the following four research questions formulated:

RQ1: What are the characteristics (descriptive) of the publications? How many papers on “mobile technologies and apps” concerning “posttraumatic stress disorder” have been published between 2010 and 2019?

RQ2: Who are the most productive authors/coauthors in this field, and what are their countries of origin? What are the citation metrics of these authors?

RQ3: In what types of sources are the papers published most frequently? Which organizations mainly contribute to this research area?

RQ4: What are the co-words (keywords/text) associated with these publications?

### Bibliometric Study

A bibliometric study enables researchers to explore patterns, trends, associations, and scientific developments related to searched domains, along with interrelated fields over publication data. This analysis requires a structured bibliometric database to obtain appropriate data for answering research questions [37-39].

Bibliometrics is also defined as a statistical method to analyze bibliometric publications data over a wide spectrum such as peer-reviewed journal articles, books, conference proceedings, periodicals, reviews, reports, and related reports. There are various analysis methods for a literature review along with bibliometric tools [37,40-43]. This approach allows for further obtaining more in-depth understanding of a given topic and its publication trends.

In this study, we employed a bibliometric approach for obtaining descriptive publication results, author/coauthor productivity metrics, source impact analysis, along with keyword and most common co-word (text) analysis [38,44-47].

### Co-word (Text) Analysis and Word Cloud

A word cloud, also known as a “tag cloud,” is a visual representation of text data [39] from various keywords or any given text material [42]. According to the Web of Science dataset structure, a word cloud has four main categories to analyze: abstract, title of the paper, author keywords, and keyword plus (see Multimedia Appendix 1). Depending on the frequency of text data regarding the main categories, the significant terms and tags are highlighted, which are usually single words represented by a single font size and color based on their relative importance. Bold and larger-sized words indicate that the word has more importance and has attracted researchers’ increasing attention in the subject domain field [48]. Keywords or any other text datasets among these four categories were collected from the articles to conduct the co-word analysis and to construct a word cloud to illustrate the power of words based on their frequencies in the literature [49].

### Data Collection and Extraction

A bibliometric study requires a structured database to analyze publication data. The main two bibliometric databases available for this purpose are ISI Web of Science and Scopus. ISI Web of Science provides data on the highest ranked and impactful (prestigious) sources, whereas Scopus also ranks the same sources in addition to other sources with wider coverage, including conferences, symposia, and congress proceedings. In addition, PubMed is a commonly used database in the medical field. Journals focused on mHealth mostly rank in the highest quartile (Q1) in Web of Science as well. Therefore, we chose to focus on these highly ranked (Q1) and high-impact sources in Web of Science to maintain consistency of citations in a single database with SCI-Expanded and SSCI indexing. We only used PubMed for comparison purposes and as a secondary source of indexing. All databases have their citation count categories. The citation results are significant to keep all publications within the same quartile and for consistency of comparisons at the same level (Q1 ranking and indexing). Finally, publication data were retrieved from the Web of Science database using the search strategy shown in Textbox 1 [9,37,38,40,42].

**Search criteria and strategy.a**

TITLE: (“*mobile*” OR “mobile*” OR “*Mobile*” OR “Mobile*”) AND

TOPIC: (“PTSD” OR “post-traumatic stress disorder” OR “post traumatic stress disorder” OR “posttraumatic stress disorder”) (Journal articles title; keywords, abstract)

Database: PubMed

Database: ISI Web of Science Core Collection (Clarivate Analytics)

Indexes: SCI-EXPANDED, SSCI

Timespan: all years of (2010-2019)

^a^64 were papers found in the Web of Science list, including 40 matching papers in PubMed.

The data were retrieved as plain .txt, .xls, .csv, and .bib file formats for further analysis. The Microsoft Excel and R Language (version R x64 3.6.1) R Studio software with the “bibliometrix” package [39] were used for descriptive and bibliometric data analysis, respectively [48].

## Results

### Publication Profile and Descriptive Publication Results

A total of 64 publications for the research domain were retrieved from the ISI Web of Science database (Multimedia Appendix 2). In comparison to the PubMed results, 40 papers were found in the same dataset of the Web of Science–retrieved publications. The vast majority of the papers were written in the English language (63/64, 98%) with the remaining article written in French (1/64, 2%). Overall, there were 323 authors/coauthors from 11 different countries, with the United States being the most common, followed by England, Canada, Italy, the Netherlands, Australia, France, Germany, Mexico, Sweden, and Vietnam. The descriptive summary of these publications showed that the majority were peer-reviewed journal articles (48/64, 75%), followed by reviews (8/64, 13%), meeting abstracts (5/64, 8%), news items (2/64, 3), and proceedings (1/64, 2%). There was a mean number of 6.4 papers published on the topic per year, with a 100% increase in the number of publications found for the period of 2016-2019, along with a corresponding increase in the mean number of papers published per year during this period to 13.33.

Web of Science-Core Collection subject category data were used to categorize the related research domains under the top 12 major subjects, which are summarized in Table 1, comprising topics with at least 3 publications. Other minor subject categories with 2 publications included Computer Science Information Systems and Neurosciences, and those with 1 paper each included Computer Science Interdisciplinary Applications, Education Educational Research, Environmental Sciences, Health Policy Services, Information Science Library Science, Medicine Research Experimental, Nursing, Oncology, Pediatrics, Psychology Experimental, Rehabilitation, and Respiratory System.

**Table 1:** Publications in the top Web of Science subject areas from 2010 to 2019 (N=64).

Subject category	Publications, n (%)
Psychiatry	23 (37)
Psychology Clinical	16 (26)
Health Care Sciences Services	11 (18)
Medical Informatics	11 (18)
Medicine General Internal	8 (13)
Psychology Multidisciplinary	6 (10)
Psychology	4 (6)
Substance Abuse	4 (6)
Clinical Neurology	3 (5)
Pharmacology Pharmacy	3 (5)
Public Environmental Occupational Health	3 (5)

### Distribution of Publications Over Time

**Figure 1:**
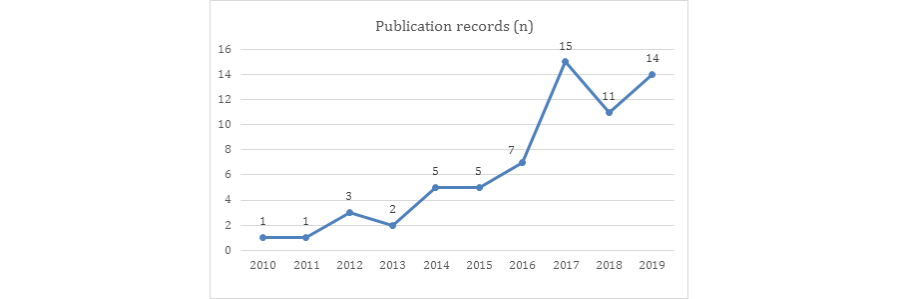
Publication records by year.

Figure 1 presents the publication records by year starting from 2010 through to the end of the decade in 2019. There was only one publication recorded for 2010 and 2011, followed by a slight increase from 2012 to 2013. From 2013 to 2014, the number of publications increased sharply from 2 to 5, with a steady increase until 2016 (n=5 to n=7). A sharp increase was observed from 2016 (n=7) to 2019 (n=14), representing a 100% increase in the publication count. The last 3 years reflect increasing research interest in the topic, as the period with the highest count in the dataset. The mean number of publications per year for the entire period was 6.4, increasing to 13.33 from 2016 to 2019. The publication distribution also supports this upward movement as shown in Figure 1.

### Most Productive Authors/Coauthors

Table 2 provides the descriptive results for authors and coauthors of the retrieved publications. Only 6 papers were published as single-authored documents, whereas 317 papers were multiple-authored documents, and there were 323 different authors with 366 appearances.

**Table 2:** Author and coauthor descriptive results for all documents published between 2010 and 2019.

Descriptive results	Value
Authors	323
Author appearances	366
Authors of single-authored documents	6
Authors of multi-authored documents	317
Documents per author	0.198
Authors per document	2.05
Coauthors per document	5.72
Collaboration index	5.56
Average citations per document	7.95

Among the top 20 most productive authors, Kuhn (n=7 records) was identified as a leading author; followed by Beckham (n=5 records); Calhoun (n=4 records); and Dennis, Marx, and Moore (n=3 records each) in the top category. Others at the same level with 2 papers each include Acierno, Carpenter MJ, Carpenter VL, Dahne, Dedert, Dennis, Diaz, Felton, Hertzberg, Hoffman, Jovanovic, Kirby, and Kizakevich.

Table 3 shows the top 10 most productive countries that contributed to the research domain fields. The top country was the United States, followed by Germany, Italy, the Netherlands, United Kingdom, Australia, Canada, France, Mexico, and Sweden.

**Table 3:** Most relevant countries by corresponding author.

Country	Articles, N	Frequency	SCP^a^	MCP^b^	MCP/article ratio
USA	45	0.776	42	3	0.07
Germany	2	0.035	2	0	0
Italy	2	0.035	2	0	0
Netherlands	2	0.035	2	0	0
United Kingdom	2	0.035	2	0	0
Australia	1	0.017	1	0	0
Canada	1	0.017	1	0	0
France	1	0.017	1	0	0
Mexico	1	0.017	1	0	0
Sweden	1	0.017	1	0	0

^a^SCP: single-country publication.

^b^MCP: multiple-country publication.

Table 4 shows the contributed institutions from various countries around the world. Harvard University and Stanford University emerged as the leading institutions with 8 papers each, and 13 universities ranked at the top of the dataset among the total 156 contributing institutions.

**Table 4:** Institutions with the greatest contributions to the field (N=156).

Institution	Publications, n (%)
Harvard University	8 (13)
Stanford University	8 (13)
Duke University	7 (11)
US Department of Veteran Affairs	7 (11)
VA Palo Alto Health Care System	7 (11)
VA Boston Healthcare System	6 (10)
Durham VA Medical Center	5 (8)
Boston University	4 (7)
Harvard Medical School	4 (7)
Medical University of South Carolina	4 (7)
University of California System	4 (7)
University of Washington	4 (7)
University of Washington Seattle	4 (7)

### Citation Results

**Figure 2:**
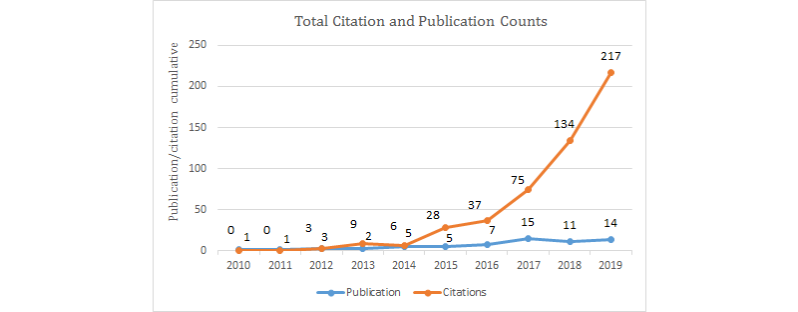
Total publication/citation counts per year (2010-2019).

The citation report of the 64 publications derived from the Web of Science Core Collection statistics between 2010 and 2019 showed that the H-index was 12 and the average number of citations per item was 7.9. The sum of times an article was cited in total was 509, which was reduced to 471 after excluding self-citations. The number of cited articles was 421 in total and was 402 after excluding self-citations. Figure 2 shows the total citation distribution throughout the research timeframe. Although the citation statistics are available as of 2012, an increase was only observed as of 2015 (n=28), followed by a steady increase until 2019. From 2016 to 2017 and from 2017 to 2018, the citation counts nearly doubled, reaching 217 by 2019.

Table 5 presents the top 10 journals by citation counts. Olff [2] was the leading paper with the highest number of citations (n=58) during the search period. The top 10 citations comprised 299 of the total 509 citations (58.7%) starting from 2012 to 2019. There was a mean of 29.9 per year. Therefore, 4 papers were above the mean: Olff [2], Hertzberg et al [19], Van Ameringen et al [50], and Repetto et al [29], published in 2015, 2013, 2017, and 2013, respectively. Although the papers only started to be cited as of 2012, the citation counts showed a substantial (3-times) increase from 2014 to 2015 (from 6 to 20 counts). The most citations were recorded in 2019 (n=108).

The leading journal publishing these studies was *Journal of Medical Internet Research (JMIR)* with 3 articles, and the remaining journals only included 1 article each published in the top 10 list. The majority of these papers were related to mobile technology and apps, PTSD-related topics, virtual reality related to mobile devices, treatment, and other psychological disorders.

**Table 5:** Top 10 articles by citation results (2010-2019).

Reference	Citations (N)									Average citations/year
	Total	2012	2013	2014	2015	2016	2017	2018	2019	
Olff [2]	58	0	0	0	8	6	17	10	17	9.67
Hertzberg et al [19]	49	0	1	2	8	7	17	8	6	6.13
Van Ameringen et al [50]	43	0	0	0	0	0	2	18	23	10.75
Repetto et al [29]	38	3	8	4	2	6	4	2	9	4.75
Place et al [51]	23	0	0	0	0	0	2	11	10	5.75
Carpenter et al [20]	22	0	0	0	2	4	6	5	5	3.67
Lui et al [3]	21	0	0	0	0	0	0	8	13	5.25
Kahn et al [5]	18	0	0	0	0	0	1	7	10	3.6
Rathbone et al [9]	14	0	0	0	0	0	0	9	5	3.5
Bakker and Rickard [52]	13	0	0	0	0	0	0	3	10	4.33
Sum	299	3	9	6	20	23	49	81	108	

### Source Frequency and Productivity

Table 6 shows the publication frequency for the most relevant sources. Although there were no notable differences in publication history, the top contributing journals of 8 sources (n≥2 records) are listed in Table 6.

The top publication sources distributed and sorted from the highest on the list with 4 (19.36%) records were *European Journal of Psychotraumatology, JMIR mHealth and uHealth,* and *Military Medicine*. The next most common sources with 3 (14.52%) records were *JMIR*, *Professional Psychology Research and Practice*, and *Psychological Services,* whereas *Applied Psychophysiology and Biofeedback* and *Depression and Anxiety* included 2 records each (6.45%).

There were 39 single-publication sources (n=1, 59.68%) among the total 64 articles in the dataset retrieved: *Addiction, Addictive Behaviors, American Journal of Preventive Medicine, Annales Medico Psychologiques, Asian Journal of Psychiatry, Behavior Therapy, Biological Psychiatry, Computers In Human Behavior, Current Psychiatry Reports, ETR D Educational Technology Research and Development, Health Informatics Journal, Implementation Science, International Journal of Clinical Practice, International Journal of Environmental Research and Public Health, International Journal of Methods In Psychiatric Research, International Journal of Psychiatry In Medicine, Jama Journal of The American Medical Association, JMIR Medical Informatics, JMIR Mental Health, Journal of Affective Disorders, Journal of Child And Adolescent Psychopharmacology, Journal of Clinical Psychiatry, Journal of Dual Diagnosis, Journal of Head Trauma Rehabilitation, Journal of Investigative Medicine, Journal of Medical Systems, Journal of Pain, Journal of Psychiatric Research, Journal of Psychosocial Nursing And Mental Health Services, Journal of The American Medical Informatics Association, Journal of Traumatic Stress, Nicotine Tobacco Research, Personal and Ubiquitous Computing, Psychiatry Interpersonal and Biological Processes, Psycho-Oncology, Social Psychiatry and Psychiatric Epidemiology, Global Mental Health, Systematic Reviews,* and *Thorax.*

**Table 6:** Most relevant top publication sources (N=64).

Source	Records, n (%)
European Journal of Psychotraumatology	4 (6)
JMIR mHealth and uHealth	4 (6)
Military Medicine	4 (6)
Journal of Medical Internet Research (JMIR)	3 (5)
Professional Psychology Research and Practice	3 (5)
Psychological Services	3 (5)
Applied Psychophysiology and Biofeedback	2 (3)
Depression and Anxiety	2 (3)

The *JMIR* group of journals had the highest amount of publication records (n=9) for the research domains in the search period. In addition, the source impact metric (H-index, n≥2) of the journals ranked as follows: *JMIR mHealth and uHealth* (H-index=4); *Military Medicine*, *JMIR*, and *Professional Psychology Research and Practice* (H-index=3); and *European Journal of Psychotraumatology, Psychological Services*, and *Depression and Anxiety* (H-index=2).

### Co-Word (Keyword) Analysis

Keyword analysis is used to reveal the frequency of keywords in publications. There are two types of keywords: author keywords and Keywords Plus. The author keywords are the words (terms) that authors prefer to use for their papers and Keyword Plus includes terms from the preset list of related science domains, which is created by the editorial experts of Web of Science.

Table 7 shows the most relevant keywords used in the publications associated with mobile technologies and apps for PTSD treatment. The most common author keywords included “m-health,” “PTSD,” “mobile health,” and “depression.” The most common Keyword Plus terms included “posttraumatic stress disorder,” “PTSD,” “randomized controlled trial,” and “depression.”

**Table 7:** Top 10 keywords.

Rank	Author keywords		Keywords-Plus	
	Term	N	Term	N
1	m-health	14	posttraumatic stress disorder	20
2	PTSD	14	PTSD	13
3	depression	9	randomized controlled trial	13
4	mobile health	7	depression	12
5	mobile apps	6	care	9
6	posttraumatic stress disorder	6	smartphone app	8
7	telemedicine	5	symptoms	8
8	trauma	5	veterans	8
9	mental health	4	meta-analysis	7
10	anxiety	3	prevalence	7

### Co-Word (Text) Analysis

**Figure 3:**
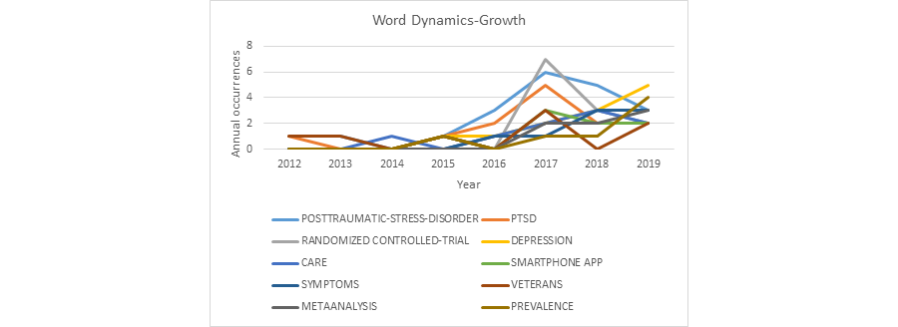
Word dynamics-growth.

The word dynamic-growth graph (Figure 3), prepared with the top keywords, was used to evaluate the keyword dynamics over the research period. The repetition trend of each word (ie, the frequency of appearances in the dataset over the search period) represents occurrences. The graph shows the trend direction to analyze either upward or downward movement over the linear line according to the annual distribution of keywords. The most popular terms and keywords can be tracked during the period to understand trends in subject domain interest and importance in the research field. Identifying topics of growing interest helps researchers to concentrate on new subject areas and can also provide valuable results to contribute to these fields.

Figure 3 illustrates the word dynamics-growth for the research domains. Although the dataset shows that publications started in 2012, the interest and popularity became significantly more visible after 2015, followed by a sharp increase in the subject areas until the middle of 2017. In those years, “PTSD,” “posttraumatic stress disorder,” “smartphone,” and “randomized controlled trial” had peak levels, but the usage rates of these terms declined from the middle of 2017 to 2019. At this point, the terms “depression” and “prevalence” became highly popular with a sharp increase on the graph, followed by “symptoms” and “meta-analysis.”

Multimedia Appendix 1 illustrates four different world cloud–based data distributions. The top left word cloud is for abstract text data, whereas the upper right word cloud is for author keywords text data. Similarly, the bottom left word cloud is from Keyword Plus text data and the bottom right word cloud shows a paper title text data representation. As can be seen in each word cloud, the highlighted words differ in terms of importance for each group. In particular, “health,” “mobile,” and “apps” were of the highest importance, followed by “mental,” “treatment,” and “PTSD” in the abstract word cloud. By contrast, in the author keyword cloud, “m-health” and “PTSD” were both dominant keywords, followed by “depression,” “mobile health,” and “posttraumatic stress disorder.” The word order changed for Keyword Plus to “depression” and “PTSD” at the top, followed by “care,” “smartphone app,” and “symptoms.” The title word cloud was comparable to the others with “mobile” being far more important than other key terms as a highly dominant term, followed by “health,” and the terms “stress,” “app,” “disorder,” “mental”, “posttraumatic,” and “application” appeared in another layer.

## Discussion

### Principal Results

Mobile apps are recognized as efficient tools in the assistance of both health care patients and staff [1,3]. A mobile platform allows for predicting the symptoms of depression and PTSD [51] to collect data and conduct research on health interventions using the technology [53]. Mobile technology in the treatment for military staff and veterans with PTSD and other mental disorders has been reported to be feasible [8,15]. Engagement with mHealth apps in the transition period improved communications [18] and mental well-being [52]. Several mobile apps have been considered to be useful supportive tools in the treatment of managing pain [26,27], stress reduction [54], anger [25], and mental disorders, including PTSD, depression, anxiety, and addictions [50,55], as well as in identifying suicidal behaviors [7].

The efficiency of mobile apps in the psychological treatment of mental disorders, including CBT [9], cognitive rehabilitation [28], exposure therapy [10,29,30], and visualization [56] has been verified. The use of mobile apps allows for evaluating CBT precision [34], to conduct a cognitive assessment [35], to deliver a PTSD checklist [36], and to monitor mental disorders [33].

By analyzing the results of the dataset regarding the publication pattern on research related to mobile technology and apps associated with PTSD treatment, we found a rapid increase and growth of subject interest in the last decade. The trend sharply increased in the most recent years, from 2016 to 2019.

This growth of productivity could reflect the improvements, functionality, and developments of mobile technology and apps in parallel with comparison to other areas of usage. Therefore, these technologies have become the center of human life to provide new opportunities, convenience, and address potential benefits. Despite the negative insights and perceptions of such technologies, this would be promising for patients to adapt their health behaviors as a supportive tool with their clinical treatments. Cooperation among clinical experts, app designers, and technology providers is necessary to reach the ultimate goal and objectives, which should concentrate on patients’ needs and treat them positively and objectively.

This analysis was conducted to highlight the most frequent subject categories, along with popular keywords and terms. These aspects were reflected in keywords, and the same terms were similarly represented in the text of the abstract and title of the paper. The text analysis showed the critical terms used in this field of research and also represents the popularity of subdomain searches. The aim of this study was to discover the publication trend and to identify the critical areas in the dataset to ultimately provide insights and research directions for academics, practitioners, and readers who wish to collaborate in these domains in the future.

According to the papers retrieved and analyzed (N=64), the majority were peer-reviewed journal articles (75%), with a mean of 6.4 publications per year from 2010 to 2016, which then sharply increased to 13.33 (doubled) between 2016 and 2019. The most productive countries were the United States, with far greater representation than any other country, followed by Germany, Italy, the Netherlands, and the United Kingdom. One of the main reasons for this difference is attributed to differences in military engagement in various regions. For example, in the Middle East and Asia, soldiers return to their countries with different intensities of PTSD symptoms and other comorbid disorders.

The results also revealed the distribution of the publications, demonstrating that the top category sources support previous arguments; namely *European Journal of Psychotraumatology, JMIR mHealth* *and uHealth*, and *Military Medicine* were among the highly popular sources for these publications. The *JMIR* group of journals emerged as the leading sources (n=9, 14% coverage) compared to other single-publication sources. However, the vast majority (60%) of sources were equally distributed among the 39 single-publication sources. Similar results were found in the authorship analysis, in which the top contributors in the field are Kuhn (n=7 records) as the leading author; followed by Beckham (n=5 records); Calhoun (n=4 records); and Dennis, Marx, and Moore (n=3 records each) in the top categories.

Co-word (text) analysis showed that the most common vital terms overall (for the four different word clouds created) were “mobile,” “PTSD,” “posttraumatic stress disorder,” “m-health,” “depression,” “health,” “treatment,” “smartphone apps,” and “mobile health.”

### Strengths and Limitations

Despite growing interest for the research domains, no publication was identified that analyzed the state of the field with a bibliometric approach. Therefore, the main strength of this study could be considered as the uniqueness of the research design itself. This study is the first bibliometric-related research in the domain. The contribution of the study is revealing the scientific patterns and future research gaps to academics and practitioners. The text analysis also highlighted and supported popular subject areas to clarify the research scope and future directions.

One of the limitations of this study is that we used only the Web of Science Core Collection database in comparison to PubMed. A single database was selected to ensure a simple and accurate analysis, and to effectively eliminate duplications and avoid errors. In this regard, Web of Science covers the highest impact journals and has unique indexing and ranking with its citation categories. The research domain of health and mobile internet–related publications is ranked in the SCI-Exp index. Another limitation is that only documents published in the English language were selected. Although various bibliometric analysis methods are available, given the scope and size of this topic, we decided to concentrate on more specific analyses such as descriptive statistics regarding the dataset from 2010 to 2019.

### Future Research Suggestions

According to the findings, the research domains are prevalent, and growing interest can be seen as an upward trend in the publication records since 2016. In particular, the majority of the subject category records were found in Psychiatry and Psychology, especially in the clinical and multidisciplinary domains, followed by Health Care Science Services, Medical Informatics, and Medicine General Internal. Further research is needed adopting various aspects of bibliometric analysis. More empirical and case studies should also be conducted in parallel with the improvement of technology and apps perspectives that would be tested and clinically validated.

However, the research analyzed indicates the importance of further explorations to develop appropriate and feasible mobile technology for PTSD treatment. The necessity to manage the challenges related to the development of mHealth tools were underlined [2]. Establishing the standards for creating PTSD-related mHealth apps and following them seem to be essential in transferring mobile apps to the clinical field [4]. Moreover, practitioners need to explore the factors facilitating and limiting the effective use of mHealth for PTSD treatment [57,58]. Cooperation between mobile app creators, researchers, and practitioners is essential in creating new technology that will match the needs and expectations of both health care staff and patients [50]. Finally, the majority of the available mobile apps require more clinically validated evidence of their efficacy before they can be adopted in the psychological treatment of PTSD [33].

### Conclusions

This study explored and analyzed the scientific patterns and relations of scholarly publications related to the use of mobile technologies in PTSD treatment. We therefore provide a general overview of the field based on co-word (text and keyword) analysis of research domains, and various forms of bibliometric methods were employed along with a data visualization approach to establish a clear picture. The analysis included 64 papers published between 2010 and 2019.

The results identify the most frequent subject categories, popular keywords, critical terms, and the popularity of subdomain searches. With this study, we attempted to investigate the patterns of publications to provide insights and research directions for academics, practitioners, and readers who wish to collaborate in these domains in the future. The data highlight the significance of further explorations in this field to improve mobile technology for PTSD treatment. Conducting studies and analyzing the practical use of these tools will improve the technology and apps that would be tested and clinically validated.

## Appendix

Multimedia Appendix 1 Word clouds visualization.

Multimedia Appendix 2 Repository dataset of publications.

## Abbreviations

CBT: cognitive behavioral therapy
mHealth: mobile health
PTSD: posttraumatic stress disorder
