# Supplementary figures and images for: Scientific Publication Patterns of Mobile Technologies and Apps for Posttraumatic Stress Disorder Treatment: Bibliometric Co-Word Analysis
Source: JMIR Mhealth Uhealth. 2020 Nov 26;8(11):e19391. doi: 10.2196/19391 (PMC7728532; doi:10.2196/19391)

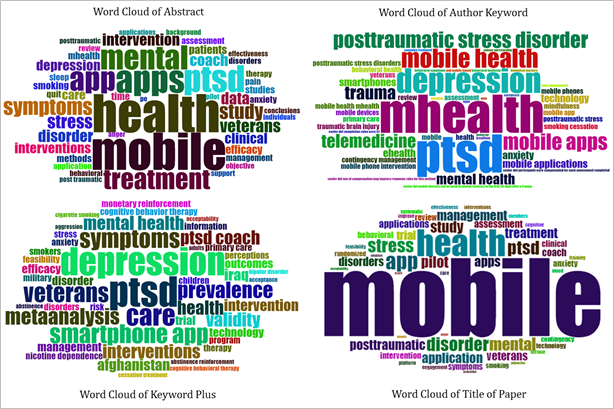

Supplement: Multimedia Appendix 1 [file mhealth_v8i11e19391_app1.png]
