# Supplementary material for: Scientific Publication Patterns of Mobile Technologies and Apps for Posttraumatic Stress Disorder Treatment: Bibliometric Co-Word Analysis
Source: JMIR Mhealth Uhealth. 2020 Nov 26;8(11):e19391. doi: 10.2196/19391 (PMC7728532; doi:10.2196/19391)
Supplement: Multimedia Appendix 2 [file mhealth_v8i11e19391_app2.pdf]

Appendix: Supplementary File (Repository Data Set of Publication List)

Web of Science

(Records 1 -- 64)

Record 1 of 64

Title: y Intervention components, mediators, and mechanisms of change of Internet- and mobile-based interventions for post-traumatic stress disorder: protocol for a systematic review and meta-analysis

Author(s): Steubl, L (Steubl, Lena); Sachser, C (Sachser, Cedric); Baumeister, H (Baumeister, Harald); Domhardt, M (Domhardt, Matthias)

Source: SYSTEMATIC REVIEWS **Volume:** 8 **Issue:** 1 **Article Number:** 265 **DOI:** 10.1186/s13643-019-1190-6 **Published:** NOV 7 2019

Language: English

Document Type: Review

Web of Science Categories: Medicine, General & Internal

eISSN: 2046-4053

Record 2 of 64

Title: Mobile telephone-delivered contingency management interventions promoting behaviour change in individuals with substance use disorders: a meta-analysis

Author(s): Getty, CA (Getty, Carol-Ann); Morande, A (Morande, Ana); Lynskey, M (Lynskey, Michael); Weaver, T (Weaver, Tim); Metrebian, N (Metrebian, Nicola)

Source: ADDICTION **Volume:** 114 **Issue:** 11 **Pages:** 1915-1925 **DOI:** 10.1111/add.14725 **Published:** NOV 2019

Language: English

Document Type: Review

Web of Science Categories: Substance Abuse; Psychiatry

ISSN: 0965-2140

eISSN: 1360-0443

Record 3 of 64

Title: Usability Testing of a Mobile Health Intervention to Address Acute Care Needs after Sexual Assault

Author(s): Gilmore, AK (Gilmore, Amanda K.); Davidson, TM (Davidson, Tatiana M.); Leone, RM (Leone, Ruschelle M.); Wray, LB (Wray, Lauren B.); Oesterle, D (Oesterle, DanielW.); Hahn, CK (Hahn, Christine K.); Flanagan, JC (Flanagan, Julianne C.); Gill-Hopple, K (Gill-Hopple, Kathleen); Acierno, R (Acierno, Ron)

Source: INTERNATIONAL JOURNAL OF ENVIRONMENTAL RESEARCH AND PUBLIC HEALTH **Volume:** 16 **Issue:** 17 **Article Number:** 3088 **DOI:** 10.3390/ijerph16173088 **Published:** SEP 1 2019

Language: English
Document Type: Article
Web of Science Categories: Environmental Sciences; Public, Environmental & Occupational Health
ISSN: 1661-7827
eISSN: 1660-4601

Record 4 of 64
Title: Assessment of users' acceptability of a mobile-based embodied conversational agent for the prevention and detection of suicidal behaviour
Author(s): Martinez-Miranda, J (Martinez-Miranda, Juan); Martinez, A (Martinez, Ariadna); Ramos, R (Ramos, Roberto); Aguilar, H (Aguilar, Hector); Jimenez, L (Jimenez, Liliana); Arias, H (Arias, Hodwar); Rosales, G (Rosales, Giovanni); Valencia, E (Valencia, Elizabeth)
Source: JOURNAL OF MEDICAL SYSTEMS **Volume:** 43 **Issue:** 8 **Article Number:** 246 **DOI:** 10.1007/s10916-019-1387-1 **Published:** AUG 2019
Language: English
Document Type: Article
Web of Science Categories: Health Care Sciences & Services; Medical Informatics
ISSN: 0148-5598
eISSN: 1573-689X

Record 5 of 64
Title: A Mobile Phone-Based Intervention to Improve Mental Health Among Homeless Young Adults: Pilot Feasibility Trial
Author(s): Schueller, SM (Schueller, Stephen M.); Glover, AC (Glover, Angela C.); Rufa, AK (Rufa, Anne K.); Dowdle, CL (Dowdle, Claire L.); Gross, GD (Gross, Gregory D.); Karnik, NS (Karnik, Niranjan S.); Zalta, AK (Zalta, Alyson K.)
Source: JMIR MHEALTH AND UHEALTH **Volume:** 7 **Issue:** 7 **Article Number:** e12347 **DOI:** 10.2196/12347 **Published:** JUL 2 2019
Language: English
Document Type: Article
Web of Science Categories: Health Care Sciences & Services; Medical Informatics
ISSN: 2291-5222

Record 6 of 64
Title: Pilot Randomized Trial of a Self-Help Behavioral Activation Mobile App for Utilization in Primary Care
Author(s): Dahne, J (Dahne, Jennifer); Lejuez, CW (Lejuez, C. W.); Diaz, VA (Diaz, Vanessa A.); Player, MS (Player, Marty S.); Kustanowitz, J (Kustanowitz, Jacob); Felton, JW (Felton, Julia W.); Carpenter, MJ (Carpenter, Matthew J.)
Source: BEHAVIOR THERAPY **Volume:** 50 **Issue:** 4 **Pages:** 817-827 **DOI:** 10.1016/j.beth.2018.12.003 **Published:** JUL 2019
Language: English

Document Type: Article
Web of Science Categories: Psychology, Clinical; Psychiatry
ISSN: 0005-7894
eISSN: 1878-1888

Record 7 of 64
Title: Characterizing the Association of Mobile Skin Conductance and Ambulatory Heart Rate on PTSD Symptom Clusters
Author(s): Trang, K (Trang, Kathy); Jovanovic, T (Jovanovic, Tanja); Worthman, C (Worthman, Carol); Hinton, D (Hinton, Devon); Sullivan, P (Sullivan, Patrick); Tran, VH (Viet Ha Tran); Go, V (Go, Vivian); Hoffman, I (Hoffman, Irving); Le, XL (Xuan Lam Le); Nguyen, KC (Kim Chi Nguyen); Le, MG (Minh Giang Le)
Source: BIOLOGICAL PSYCHIATRY **Meeting Abstract:** S24 **Volume:** 85 **Issue:** 10 **Pages:** S305-S306 **DOI:** 10.1016/j.biopsych.2019.03.775 **Supplement:** S **Published:** MAY 15 2019
Language: English
Document Type: Meeting Abstract
Conference Title: 74th Annual Meeting of the Society-of-Biological-Psychiatry (SOBP)
Conference Date: MAY 16-18, 2019
Conference Location: Chicago, IL
Web of Science Categories: Neurosciences; Psychiatry
ISSN: 0006-3223
eISSN: 1873-2402

Record 8 of 64
Title: Mobile App Interventions for Military and Veteran Families: Before, During, and After Deployment
Author(s): Nolan, J (Nolan, Jon); Lindeman, S (Lindeman, Sarah); Varghese, FP (Varghese, Femina P.)
Source: PSYCHOLOGICAL SERVICES **Volume:** 16 **Issue:** 2 **Special Issue:** SI **Pages:** 208-212 **DOI:** 10.1037/ser0000272 **Published:** MAY 2019
Language: English
Document Type: Article
Web of Science Categories: Psychology, Clinical
ISSN: 1541-1559
eISSN: 1939-148X

Record 9 of 64
Title: PTSD Coach Mobile Application With Brief Telephone Support: A Pilot Study
Author(s): Tiet, QQ (Tiet, Quyen Q.); Duong, H (Duong, Heather); Davis, L (Davis, Laila); French, R (French, Rebecca); Smith, CL (Smith, Christopher L.); Leyva, YE (Leyva, Yani E.); Rosen, C (Rosen, Craig)

Source: PSYCHOLOGICAL SERVICES **Volume:** 16 **Issue:** 2 **Special Issue:** SI **Pages:** 227-232 **DOI:** 10.1037/ser0000245 **Published:** MAY 2019

Language: English

Document Type: Article

Web of Science Categories: Psychology, Clinical

ISSN: 1541-1559

eISSN: 1939-148X

Record 10 of 64

Title: A Randomized Controlled Trial of the PTSD Coach Mobile Health App at Reducing Pain and Psychological Symptoms among Injured Emergency Department Patients: Preliminary Results

Author(s): Pacella, M (Pacella, M.); Germain, A (Germain, A.); Suffoletto, B (Suffoletto, B.); Kuhn, E (Kuhn, E.); Jaramillo, S (Jaramillo, S.); Caaway, C (Caaway, C.)

Source: JOURNAL OF PAIN **Meeting Abstract:** 148 **Volume:** 20 **Issue:** 4 **Pages:** S13-S13 **DOI:** 10.1016/j.jpain.2019.01.067 **Supplement:** 1 **Published:** APR 2019

Language: English

Document Type: Meeting Abstract

Web of Science Categories: Clinical Neurology; Neurosciences

ISSN: 1526-5900

Record 11 of 64

Title: Adoption of Mobile Apps for Depression and Anxiety: Cross-Sectional Survey Study on Patient Interest and Barriers to Engagement

Author(s): Lipschitz, J (Lipschitz, Jessica); Miller, CJ (Miller, Christopher J.); Hogan, TP (Hogan, Timothy P.); Burdick, KE (Burdick, Katherine E.); Lippin-Foster, R (Lippin-Foster, Rachel); Simon, SR (Simon, Steven R.); Burgess, J (Burgess, James)

Source: JMIR MENTAL HEALTH **Volume:** 6 **Issue:** 1 **Article Number:** PMID 30681968 **DOI:** 10.2196/11334 **Published:** JAN 25 2019

Language: English

Document Type: Article

Web of Science Categories: Psychiatry

ISSN: 2368-7959

Record 12 of 64

Title: Proposing a standardized, step-by-step model for creating post-traumatic stress disorder (PTSD) related mobile mental health apps in a framework based on technical and medical norms

Author(s): Schellong, J (Schellong, Julia); Lorenz, P (Lorenz, Patrick); Weidner, K (Weidner, Kerstin)

Source: EUROPEAN JOURNAL OF PSYCHOTRAUMATOLOGY **Volume:** 10 **Issue:** 1 **Article Number:** 1611090 **DOI:** 10.1080/20008198.2019.1611090 **Published:** JAN 1 2019

Language: English

Document Type: Article

Web of Science Categories: Psychology, Clinical; Psychiatry

ISSN: 2000-8198
eISSN: 2000-8066

Record 13 of 64
Title: Cognitive Rehabilitation With Mobile Technology and Social Support for Veterans With TBI and PTSD: A Randomized Clinical Trial
Author(s): Elbogen, EB (Elbogen, Eric B.); Dennis, PA (Dennis, Paul A.); Van Voorhees, EE (Van Voorhees, Elizabeth E.); Blakey, SM (Blakey, Shannon M.); Johnson, JL (Johnson, Jacqueline L.); Johnson, SC (Johnson, Sally C.); Wagner, HR (Wagner, H. Ryan); Hamer, RM (Hamer, Robert M.); Beckham, JC (Beckham, Jean C.); Manly, T (Manly, Tom); Belger, A (Belger, Aysenil)
Source: JOURNAL OF HEAD TRAUMA REHABILITATION **Volume:** 34 **Issue:** 1 **Special Issue:** SI **Pages:** 1-10 **DOI:** 10.1097/HTR.0000000000000435 **Published:** JAN-FEB 2019
Language: English
Document Type: Article
Web of Science Categories: Clinical Neurology; Rehabilitation
ISSN: 0885-9701
eISSN: 1550-509X

Record 14 of 64
Title: Effects of mindfulness training programmes delivered by a self-directed mobile app and by telephone compared with an education programme for survivors of critical illness: a pilot randomised clinical trial
Author(s): Cox, CE (Cox, Christopher E.); Hough, CL (Hough, Catherine L.); Jones, DM (Jones, Derek M.); Ungar, A (Ungar, Anna); Reagan, W (Reagan, Wen); Key, MD (Key, Mary D.); Gremore, T (Gremore, Tina); Olsen, MK (Olsen, Maren K.); Sanders, L (Sanders, Linda); Greeson, JM (Greeson, Jeffrey M.); Porter, LS (Porter, Laura S.)
Source: THORAX **Volume:** 74 **Issue:** 1 **Pages:** 33-42 **DOI:** 10.1136/thoraxjnl-2017-211264 **Published:** JAN 2019
Language: English
Document Type: Article
Web of Science Categories: Respiratory System
ISSN: 0040-6376
eISSN: 1468-3296

Record 15 of 64
Title: A systematic review of the effectiveness of mobile apps for monitoring and management of mental health symptoms or disorders
Author(s): Wang, K (Wang, Kai); Varma, DS (Varma, Deepthi S.); Prosperi, M (Prosperi, Mattia)
Source: JOURNAL OF PSYCHIATRIC RESEARCH **Volume:** 107 **Pages:** 73-78 **DOI:** 10.1016/j.jpsychires.2018.10.006 **Published:** DEC 2018
Language: English
Document Type: Review

Web of Science Categories: Psychiatry
ISSN: 0022-3956
eISSN: 1879-1379

Record 16 of 64

Title: A pilot study of user satisfaction and perceived helpfulness of the Swedish version of the mobile app PTSD Coach

Author(s): Cernvall, M (Cernvall, Martin); Sveen, J (Sveen, Josefin); Johannesson, KB (Johannesson, Kerstin Bergh); Arnberg, F (Arnberg, Filip)

Source: EUROPEAN JOURNAL OF PSYCHOTRAUMATOLOGY **Volume:** 9 **Special Issue:** SI **Article Number:** 1472990 **DOI:** 10.1080/20008198.2018.1472990 **Supplement:** 1 **Published:** NOV 1 2018

Language: English

Document Type: Article

Web of Science Categories: Psychology, Clinical; Psychiatry

ISSN: 2000-8198

eISSN: 2000-8066

Record 17 of 64

Title: Implementing assessments via mobile during the acute posttrauma period: feasibility, acceptability and strategies to improve response rates

Author(s): Price, M (Price, Matthew); Van Stolk-Cooke, K (Van Stolk-Cooke, Katherine); Legrand, AC (Legrand, Alison C.); Brier, ZMF (Brier, Zoe M. F.); Ward, HL (Ward, Hannah L.); Connor, JP (Connor, Julie P.); Gratton, J (Gratton, Jennifer); Freeman, K (Freeman, Kalev); Skalka, C (Skalka, Christian)

Source: EUROPEAN JOURNAL OF PSYCHOTRAUMATOLOGY **Volume:** 9 **Special Issue:** SI **DOI:** 10.1080/20008198.2018.1500822 **Supplement:** 1 **Published:** NOV 1 2018

Language: English

Document Type: Article

Web of Science Categories: Psychology, Clinical; Psychiatry

ISSN: 2000-8198

eISSN: 2000-8066

Record 18 of 64

Title: Leveraging routine clinical materials and mobile technology to assess CBT fidelity: the Innovative Methods to Assess Psychotherapy Practices (imAPP) study

Author(s): Stirman, SW (Stirman, Shannon Wiltsey); Marques, L (Marques, Luana); Creed, TA (Creed, Torrey A.); Gutner, CA (Gutner, Cassidy A.); DeRubeis, R (DeRubeis, Robert); Barnett, PG (Barnett, Paul G.); Kuhn, E (Kuhn, Eric); Suvak, M (Suvak, Michael); Owen, J (Owen, Jason); Vogt, D (Vogt, Dawne); Jo, B (Jo, Booil); Schoenwald, S (Schoenwald, Sonja); Johnson, C (Johnson, Clara); Mallard, K (Mallard, Kera); Beristianos, M (Beristianos, Matthew); La Bash, H (La Bash, Heidi)

Source: IMPLEMENTATION SCIENCE **Volume:** 13 **Article Number:** 69 **DOI:** 10.1186/s13012-018-0756-3 **Published:** MAY 22 2018

Language: English
Document Type: Article
Web of Science Categories: Health Care Sciences & Services; Health Policy & Services
ISSN: 1748-5908

Record 19 of 64
Title: An Evaluation of Mobile Applications Designed to Assist Service Members and Veterans Transitioning to Civilian Life
Author(s): Fraynt, R (Fraynt, Rebecca); Cooper, D (Cooper, David); Edwards-Stewart, A (Edwards-Stewart, Amanda); Hoyt, T (Hoyt, Tim); Micheel, L (Micheel, Logan); Pruitt, L (Pruitt, Larry); Skopp, N (Skopp, Nancy); Smolenski, D (Smolenski, Derek)
Source: PSYCHOLOGICAL SERVICES **Volume:** 15 **Issue:** 2 **Special Issue:** SI **Pages:** 208-215 **DOI:** 10.1037/ser0000205 **Published:** MAY 2018
Language: English
Document Type: Article
Web of Science Categories: Psychology, Clinical
ISSN: 1541-1559
eISSN: 1939-148X

Record 20 of 64
Title: Mobile contingency management as an adjunctive treatment for co-morbid cannabis use disorder and cigarette smoking
Author(s): Beckham, JC (Beckham, Jean C.); Adkisson, KA (Adkisson, Kelsie A.); Hertzberg, J (Hertzberg, Jeffrey); Kimbrel, NA (Kimbrel, Nathan A.); Budney, AJ (Budney, Alan J.); Stephens, RS (Stephens, Robert S.); Moore, SD (Moore, Scott D.); Calhoun, PS (Calhoun, Patrick S.)
Source: ADDICTIVE BEHAVIORS **Volume:** 79 **Pages:** 86-92 **DOI:** 10.1016/j.addbeh.2017.12.007 **Published:** APR 2018
Language: English
Document Type: Article
Web of Science Categories: Psychology, Clinical; Substance Abuse
ISSN: 0306-4603
eISSN: 1873-6327

Record 21 of 64
Title: PHIT for Duty, a Mobile Application for Stress Reduction, Sleep Improvement, and Alcohol Moderation
Author(s): Kizakevich, PN (Kizakevich, Paul N.); Eckhoff, R (Eckhoff, Randall); Brown, J (Brown, Janice); Tueller, SJ (Tueller, Stephen J.); Weimer, B (Weimer, Belinda); Bell, S (Bell, Stacey); Weeks, A (Weeks, Adam); Hourani, LL (Hourani, Laurel L.); Spira, JL (Spira, James L.); King, LA (King, Laurel A.)
Source: MILITARY MEDICINE **Volume:** 183 **Pages:** 353-363 **DOI:** 10.1093/milmed/usx157 **Supplement:** S **Published:** MAR-APR 2018

Language: English
Document Type: Article; Proceedings Paper
Conference Title: Military Health System Research Symposium (MHSRS)
Conference Date: 2016
Conference Location: FL
Web of Science Categories: Medicine, General & Internal
ISSN: 0026-4075
eISSN: 1930-613X

Record 22 of 64
Title: Engagement in mobile phone app for self-monitoring of emotional wellbeing predicts changes in mental health: MoodPrism
Author(s): Bakker, D (Bakker, David); Rickard, N (Rickard, Nikki)
Source: JOURNAL OF AFFECTIVE DISORDERS **Volume:** 227 **Pages:** 432-442 **DOI:** 10.1016/j.jad.2017.11.016 **Published:** FEB 2018
Language: English
Document Type: Article
Web of Science Categories: Clinical Neurology; Psychiatry
ISSN: 0165-0327
eISSN: 1573-2517

Record 23 of 64
Title: The utility and feasibility of assessing sleep disruption in a men's health clinic using a mobile health platform device: A pilot study
Author(s): Le, HH (Le, Hai H.); Salas, RME (Salas, Rachel Marie E.); Gamaldo, A (Gamaldo, Alyssa); Billups, KL (Billups, Kevin L.); Dziedzic, P (Dziedzic, Peter); Choi, S (Choi, Seulah); Bermudez, N (Bermudez, Neftali); Thorpe, RJ (Thorpe, Roland J.); Gamaldo, CE (Gamaldo, Charlene E.)
Source: INTERNATIONAL JOURNAL OF CLINICAL PRACTICE **Volume:** 72 **Issue:** 1 **Article Number:** e12999 **DOI:** 10.1111/ijcp.12999 **Published:** JAN 2018
Language: English
Document Type: Article
Web of Science Categories: Medicine, General & Internal; Pharmacology & Pharmacy
ISSN: 1368-5031
eISSN: 1742-1241

Record 24 of 64
Title: Cancer distress coach: Pilot study of a mobile app for managing posttraumatic stress
Author(s): Smith, SK (Smith, Sophia K.); Kuhn, E (Kuhn, Eric); O'Donnell, J (O'Donnell, Jonathan); Koontz, BF (Koontz, Bridget F.); Nelson, N (Nelson, Nicole); Molloy, K (Molloy, Kiera); Chang, JH (Chang, Jianhong); Hoffman, J (Hoffman, Julia)
Source: PSYCHO-ONCOLOGY **Volume:** 27 **Issue:** 1 **Pages:** 350-353 **DOI:** 10.1002/pon.4363 **Published:** JAN 2018

Language: English
Document Type: Article
Web of Science Categories: Oncology; Psychology; Psychology, Multidisciplinary; Social Sciences, Biomedical
ISSN: 1057-9249
eISSN: 1099-1611

Record 25 of 64
Title: Mobile Technology for Treatment Augmentation in Veteran Smokers With Posttraumatic Stress Disorder
Author(s): Herbst, E (Herbst, Ellen); Pennington, D (Pennington, David); Kuhn, E (Kuhn, Eric); McCaslin, SE (McCaslin, Shannon E.); Delucchi, K (Delucchi, Kevin); Batki, SL (Batki, Steven L.); Dickter, B (Dickter, Benjamin); Carmody, T (Carmody, Timothy)
Source: AMERICAN JOURNAL OF PREVENTIVE MEDICINE **Volume:** 54 **Issue:** 1 **Pages:** 124-128 **DOI:** 10.1016/j.amepre.2017.08.016 **Published:** JAN 2018
Language: English
Document Type: Article
Web of Science Categories: Public, Environmental & Occupational Health; Medicine, General & Internal
ISSN: 0749-3797
eISSN: 1873-2607

Record 26 of 64
Title: Barriers and Facilitators to Mobile Application Use During PTSD Treatment: Clinician Adoption of PE Coach
Author(s): Reger, GM (Reger, Greg M.); Browne, KC (Browne, Kendall C.); Campellone, TR (Campellone, Timothy R.); Simons, C (Simons, Carol); Kuhn, E (Kuhn, Eric); Fortney, JC (Fortney, John C.); Sayre, GG (Sayre, George G.); Reisinger, HS (Reisinger, Heather Schacht)
Source: PROFESSIONAL PSYCHOLOGY-RESEARCH AND PRACTICE **Volume:** 48 **Issue:** 6 **Pages:** 510-517 **DOI:** 10.1037/pro0000153 **Published:** DEC 2017
Language: English
Document Type: Article
Web of Science Categories: Psychology, Multidisciplinary
ISSN: 0735-7028
eISSN: 1939-1323

Record 27 of 64
Title: Using a Mobile Application in the Treatment of Dysregulated Anger Among Veterans
Author(s): Mackintosh, MA (Mackintosh, Margaret-Anne); Niehaus, J (Niehaus, James); Taft, CT (Taft, Casey T.); Marx, BP (Marx, Brian P.); Grubbs, K (Grubbs, Kathleen); Morland, LA (Morland, Leslie A.)

Source: MILITARY MEDICINE **Volume:** 182 **Issue:** 11-12 **Pages:** E1941-E1949 **DOI:** 10.7205/MILMED-D-17-00063 **Published:** NOV-DEC 2017
Language: English
Document Type: Article
Web of Science Categories: Medicine, General & Internal
ISSN: 0026-4075
eISSN: 1930-613X

Record 28 of 64
Title: Assessing the Efficacy of Mobile Health Apps Using the Basic Principles of Cognitive Behavioral Therapy: Systematic Review
Author(s): Rathbone, AL (Rathbone, Amy Leigh); Clarry, L (Clarry, Laura); Prescott, J (Prescott, Julie)
Source: JOURNAL OF MEDICAL INTERNET RESEARCH **Volume:** 19 **Issue:** 11 **Article Number:** e399 **DOI:** 10.2196/jmir.8598 **Published:** NOV 2017
Language: English
Document Type: Review
Web of Science Categories: Health Care Sciences & Services; Medical Informatics
ISSN: 1438-8871

Record 29 of 64
Title: The Use of Telemedicine and Mobile Technology to Promote Population Health and Population Management for Psychiatric Disorders
Author(s): Turvey, C (Turvey, Carolyn); Fortney, J (Fortney, John)
Source: CURRENT PSYCHIATRY REPORTS **Volume:** 19 **Issue:** 11 **Article Number:** 88 **DOI:** 10.1007/s11920-017-0844-0 **Published:** NOV 2017
Language: English
Document Type: Review
Web of Science Categories: Psychiatry
ISSN: 1523-3812
eISSN: 1535-1645

Record 30 of 64
Title: Posttraumatic Stress Disorder and Mobile Health: App Investigation and Scoping Literature Review
Author(s): Rodriguez-Paras, C (Rodriguez-Paras, Carolina); Tippey, K (Tippey, Kathryn); Brown, E (Brown, Elaine); Sasangohar, F (Sasangohar, Farzan); Creech, S (Creech, Suzannah); Kum, HC (Kum, Hye-Chung); Lawley, M (Lawley, Mark); Benzer, JK (Benzer, Justin K.)
Source: JMIR MHEALTH AND UHEALTH **Volume:** 5 **Issue:** 10 **Article Number:** e156 **DOI:** 10.2196/mhealth.7318 **Published:** OCT 2017
Language: English
Document Type: Review
Web of Science Categories: Health Care Sciences & Services; Medical Informatics

ISSN: 2291-5222

Record 31 of 64

Title: Pain Self-Management for Veterans: Development and Pilot Test of a Stage-Based Mobile-Optimized Intervention

Author(s): Johnson, SS (Johnson, Sara S.); Levesque, DA (Levesque, Deborah A.); Broderick, LE (Broderick, Lynne E.); Bailey, DG (Bailey, Dustin G.); Kerns, RD (Kerns, Robert D.)

Source: JMIR MEDICAL INFORMATICS **Volume:** 5 **Issue:** 4 **Article Number:** e40 **DOI:** 10.2196/medinform.7117 **Published:** OCT-DEC 2017

Language: English

Document Type: Article

Web of Science Categories: Medical Informatics

eISSN: 2291-9694

Record 32 of 64

Title: Screening for trauma-related symptoms via a smartphone app: The validity of Smart Assessment on your Mobile in referred police officers

Author(s): van der Meer, CAI (van der Meer, Christianne A. I.); Bakker, A (Bakker, Anne); Schrieken, BAL (Schrieken, Bart A. L.); Hoofwijk, MC (Hoofwijk, Marthe C.); Olff, M (Olff, Miranda)

Source: INTERNATIONAL JOURNAL OF METHODS IN PSYCHIATRIC RESEARCH **Volume:** 26 **Issue:** 3 **Article Number:** e1579 **DOI:** 10.1002/mpr.1579 **Published:** SEP 2017

Language: English

Document Type: Article

Web of Science Categories: Psychiatry

ISSN: 1049-8931

eISSN: 1557-0657

Record 33 of 64

Title: Internet and mobile technologies: addressing the mental health of trauma survivors in less resourced communities

Author(s): Ruzek, JI (Ruzek, J. I.); Yeager, CM (Yeager, C. M.)

Source: GLOBAL MENTAL HEALTH **Volume:** 4 **Article Number:** e16 **DOI:** 10.1017/gmh.2017.11 **Published:** AUG 30 2017

Language: English

Document Type: Review

Web of Science Categories: Psychiatry

ISSN: 2054-4251

Record 34 of 64

Title: Evidence-Based Apps? A Review of Mental Health Mobile Applications in a Psychotherapy Context

Author(s): Lui, JHL (Lui, Joyce H. L.); Marcus, DK (Marcus, David K.); Barry, CT (Barry, Christopher T.)
Source: PROFESSIONAL PSYCHOLOGY-RESEARCH AND PRACTICE **Volume:** 48 **Issue:** 3 **DOI:** 10.1037/pro0000122 **Published:** JUN 2017
Language: English
Document Type: Review
Web of Science Categories: Psychology, Multidisciplinary
ISSN: 0735-7028
eISSN: 1939-1323

Record 35 of 64
Title: Mobile assessment of heightened skin conductance in posttraumatic stress disorder
Author(s): Hinrichs, R (Hinrichs, Rebecca); Michopoulos, V (Michopoulos, Vasiliki); Winters, S (Winters, Sterling); Rothbaum, AO (Rothbaum, Alex O.); Rothbaum, BO (Rothbaum, Barbara O.); Ressler, KJ (Ressler, Kerry J.); Jovanovic, T (Jovanovic, Tanja)
Source: DEPRESSION AND ANXIETY **Volume:** 34 **Issue:** 6 **Special Issue:** SI **Pages:** 502-507 **DOI:** 10.1002/da.22610 **Published:** JUN 2017
Language: English
Document Type: Article
Web of Science Categories: Psychology, Clinical; Psychiatry; Psychology
ISSN: 1091-4269
eISSN: 1520-6394

Record 36 of 64
Title: There is an app for that! The current state of mobile applications (apps) for DSM-5 obsessive-compulsive disorder, posttraumatic stress disorder, anxiety and mood disorders
Author(s): Van Ameringen, M (Van Ameringen, Michael); Turna, J (Turna, Jasmine); Khalesi, Z (Khalesi, Zahra); Pullia, K (Pullia, Katrina); Patterson, B (Patterson, Beth)
Source: DEPRESSION AND ANXIETY **Volume:** 34 **Issue:** 6 **Special Issue:** SI **Pages:** 526-539 **DOI:** 10.1002/da.22657 **Published:** JUN 2017
Language: English
Document Type: Review
Web of Science Categories: Psychology, Clinical; Psychiatry; Psychology
ISSN: 1091-4269
eISSN: 1520-6394

Record 37 of 64
Title: Behavioral Indicators on a Mobile Sensing Platform Predict Clinically Validated Psychiatric Symptoms of Mood and Anxiety Disorders
Author(s): Place, S (Place, Skyler); Blanch-Hartigan, D (Blanch-Hartigan, Danielle); Rubin, C (Rubin, Channah); Gorrostieta, C (Gorrostieta, Cristina); Mead, C (Mead, Caroline); Kane, J (Kane, John); Marx, BP (Marx, Brian P.); Feast, J (Feast, Joshua); Deckersbach, T (Deckersbach, Thilo); Pentland, A (Pentland, Alex Sandy); Nierenberg, A (Nierenberg, Andrew); Azarbayejani, A (Azarbayejani, Ali)

Source: JOURNAL OF MEDICAL INTERNET RESEARCH **Volume:** 19 **Issue:** 3 **Article Number:** e75 **DOI:** 10.2196/jmir.6678 **Published:** MAR 2017

Language: English

Document Type: Article

Web of Science Categories: Health Care Sciences & Services; Medical Informatics

ISSN: 1438-8871

Record 38 of 64

Title: Moodivate: A self-help behavioral activation mobile app for utilization in primary care-Development and clinical considerations

Author(s): Dahne, J (Dahne, Jennifer); Lejuez, CW (Lejuez, C. W.); Kustanowitz, J (Kustanowitz, Jacob); Felton, JW (Felton, Julia W.); Diaz, VA (Diaz, Vanessa A.); Player, MS (Player, Marty S.); Carpenter, MJ (Carpenter, Matthew J.)

Source: INTERNATIONAL JOURNAL OF PSYCHIATRY IN MEDICINE **Volume:** 52 **Issue:** 2 **Pages:** 160-175 **DOI:** 10.1177/0091217417720899 **Published:** MAR 2017

Language: English

Document Type: Article

Web of Science Categories: Psychiatry

ISSN: 0091-2174

eISSN: 1541-3527

Record 39 of 64

Title: A Preliminary Investigation of a Relapse Prevention Mobile Application to Maintain Smoking Abstinence Among Individuals With Posttraumatic Stress Disorder

Author(s): Hicks, TA (Hicks, Terrell A.); Thomas, SP (Thomas, Shaun P.); Wilson, SM (Wilson, Sarah M.); Calhoun, PS (Calhoun, Patrick S.); Kuhn, ER (Kuhn, Eric R.); Beckham, JC (Beckham, Jean C.)

Source: JOURNAL OF DUAL DIAGNOSIS **Volume:** 13 **Issue:** 1 **Pages:** 15-20 **DOI:** 10.1080/15504263.2016.1267828 **Published:** 2017

Language: English

Document Type: Article

Web of Science Categories: Psychology, Clinical; Substance Abuse; Psychiatry

ISSN: 1550-4263

eISSN: 1550-4271

Record 40 of 64

Title: Facilitating mental health screening of war-torn populations using mobile applications

Author(s): Hashemi, B (Hashemi, Bahar); Ali, S (Ali, Sara); Awaad, R (Awaad, Rania); Soudi, L (Soudi, Laila); Housel, L (Housel, Lawrence); Sosebee, SJ (Sosebee, Stephen J.)

Source: SOCIAL PSYCHIATRY AND PSYCHIATRIC EPIDEMIOLOGY **Volume:** 52 **Issue:** 1 **Pages:** 27-33 **DOI:** 10.1007/s00127-016-1303-7 **Published:** JAN 2017

Language: English

Document Type: Article

Web of Science Categories: Psychiatry
ISSN: 0933-7954
eISSN: 1433-9285

Record 41 of 64
Title: Mobile Applications for Mental Health Providers
Author(s): Morganstein, J (Morganstein, Joshua)
Source: PSYCHIATRY-INTERPERSONAL AND BIOLOGICAL PROCESSES **Volume:** 79 **Issue:** 4 **Pages:** 358-363 **DOI:** 10.1080/00332747.2016.1237753 **Published:** WIN 2016
Language: English
Document Type: Article
Web of Science Categories: Psychiatry
ISSN: 0033-2747
eISSN: 1943-281X

Record 42 of 64
Title: Using a Mobile Application in the Management of Anger Problems Among Veterans: A Pilot Study
Author(s): Morland, LA (Morland, Leslie A.); Niehaus, J (Niehaus, James); Taft, C (Taft, Casey); Marx, BP (Marx, Brian P.); Menez, U (Menez, Ursula); Mackintosh, MA (Mackintosh, Margaret-Anne)
Source: MILITARY MEDICINE **Volume:** 181 **Issue:** 9 **Pages:** 990-995 **DOI:** 10.7205/MILMED-D-15-00293 **Published:** SEP 2016
Language: English
Document Type: Article
Web of Science Categories: Medicine, General & Internal
ISSN: 0026-4075
eISSN: 1930-613X

Record 43 of 64
Title: Post-9/11 Veterans and Their Partners Improve Mental Health Outcomes with a Self-directed Mobile and Web-based Wellness Training Program: A Randomized Controlled Trial
Author(s): Kahn, JR (Kahn, Janet R.); Collinge, W (Collinge, William); Soltysik, R (Soltysik, Robert)
Source: JOURNAL OF MEDICAL INTERNET RESEARCH **Volume:** 18 **Issue:** 9 **Article Number:** e255 **DOI:** 10.2196/jmir.5800 **Published:** SEP 2016
Language: English
Document Type: Article
Web of Science Categories: Health Care Sciences & Services; Medical Informatics
ISSN: 1438-8871

Record 44 of 64
Title: Disaster psychiatry in Asia: The potential of smartphones, mobile, and connected technologies
Author(s): Sobowale, K (Sobowale, Kunmi); Torous, J (Torous, John)
Source: ASIAN JOURNAL OF PSYCHIATRY **Volume:** 22 **Pages:** 1-5 **DOI:** 10.1016/j.ajp.2016.03.004 **Published:** AUG 2016
Language: English
Document Type: Article
Web of Science Categories: Psychiatry
ISSN: 1876-2018
eISSN: 1876-2026

Record 45 of 64
Title: Variations in the Use of mHealth Tools: The VA Mobile Health Study
Author(s): Frisbee, KL (Frisbee, Kathleen L.)
Source: JMIR MHEALTH AND UHEALTH **Volume:** 4 **Issue:** 3 **Article Number:** e89 **DOI:** 10.2196/mhealth.3726 **Published:** JUL-SEP 2016
Language: English
Document Type: Article
Web of Science Categories: Health Care Sciences & Services; Medical Informatics
ISSN: 2291-5222

Record 46 of 64
Title: Mobile Device-Based Applications for Childhood Anxiety Disorders
Author(s): Whiteside, SPH (Whiteside, Stephen P. H.)
Source: JOURNAL OF CHILD AND ADOLESCENT PSYCHOPHARMACOLOGY **Volume:** 26 **Issue:** 3 **Pages:** 246-251 **DOI:** 10.1089/cap.2015.0010 **Published:** APR 2016
Language: English
Document Type: Article
Web of Science Categories: Pediatrics; Pharmacology & Pharmacy; Psychiatry
ISSN: 1044-5463
eISSN: 1557-8992

Record 47 of 64
Title: Assessment of patient engagement with a mobile application among service members in transition
Author(s): Pavliscsak, H (Pavliscsak, Holly); Little, JR (Little, Jeanette R.); Poropatich, RK (Poropatich, Ronald K.); McVeigh, FL (McVeigh, Francis L.); Tong, J (Tong, James); Tillman, JS (Tillman, Johnie S.); Smith, CH (Smith, Challis H.); Fonda, SJ (Fonda, Stephanie Jo)

Source: JOURNAL OF THE AMERICAN MEDICAL INFORMATICS ASSOCIATION **Volume:** 23 **Issue:** 1 **Pages:** 110-118 **DOI:** 10.1093/jamia/ocv121 **Published:** JAN 2016

Language: English

Document Type: Article

Web of Science Categories: Computer Science, Information Systems; Computer Science, Interdisciplinary Applications; Health Care Sciences & Services; Information Science & Library Science; Medical Informatics

ISSN: 1067-5027

eISSN: 1527-974X

Record 48 of 64

Title: Clinician Characteristics and Perceptions Related to Use of the PE (Prolonged Exposure) Coach Mobile App

Author(s): Kuhn, E (Kuhn, Eric); Crowley, JJ (Crowley, Jill J.); Hoffman, JE (Hoffman, Julia E.); Eftekhari, A (Eftekhari, Afsoon); Ramsey, KM (Ramsey, Kelly M.); Owen, JE (Owen, Jason E.); Reger, GM (Reger, Greg M.); Ruzek, JI (Ruzek, Josef I.)

Source: PROFESSIONAL PSYCHOLOGY-RESEARCH AND PRACTICE **Volume:** 46 **Issue:** 6 **Pages:** 437-443 **DOI:** 10.1037/pro0000051 **Published:** DEC 2015

Language: English

Document Type: Article

Web of Science Categories: Psychology, Multidisciplinary

ISSN: 0735-7028

eISSN: 1939-1323

Record 49 of 64

Title: Comparison of the PTSD Checklist (PCL) Administered via a Mobile Device Relative to a Paper Form

Author(s): Price, M (Price, Matthew); Kuhn, E (Kuhn, Eric); Hoffman, JE (Hoffman, Julia E.); Ruzek, J (Ruzek, Josef); Acierno, R (Acierno, Ron)

Source: JOURNAL OF TRAUMATIC STRESS **Volume:** 28 **Issue:** 5 **Pages:** 480-483 **DOI:** 10.1002/jts.22037 **Published:** OCT 2015

Language: English

Document Type: Article

Web of Science Categories: Psychology, Clinical; Psychiatry

ISSN: 0894-9867

eISSN: 1573-6598

Record 50 of 64

Title: Multicomponent Smoking Cessation Treatment Including Mobile Contingency Management in Homeless Veterans

Author(s): Carpenter, VL (Carpenter, Vickie L.); Hertzberg, JS (Hertzberg, Jeffrey S.); Kirby, AC (Kirby, Angela C.); Calhoun, PS (Calhoun, Patrick S.); Moore, SD (Moore, Scott D.); Dennis, MF (Dennis, Michelle F.); Dennis, PA (Dennis, Paul A.); Dedert, EA (Dedert, Eric A.); Hair, LP (Hair, Lauren P.); Beckham, JC (Beckham, Jean C.)

Source: JOURNAL OF CLINICAL PSYCHIATRY **Volume:** 76 **Issue:** 7 **Pages:** 959-964 **DOI:** 10.4088/JCP.14m09053 **Published:** JUL 2015

Language: English

Document Type: Article

Web of Science Categories: Psychology, Clinical; Psychiatry

ISSN: 0160-6689

eISSN: 1555-2101

Record 51 of 64

Title: Mobile mental health: a challenging research agenda

Author(s): Olff, M (Olff, Miranda)

Source: EUROPEAN JOURNAL OF PSYCHOTRAUMATOLOGY **Volume:** 6 **Special Issue:** SI **Article Number:** 27882 **DOI:** 10.3402/ejpt.v6.27882 **Published:** MAY 19 2015

Language: English

Document Type: Article

Web of Science Categories: Psychology, Clinical; Psychiatry

ISSN: 2000-8198

eISSN: 2000-8066

Record 52 of 64

Title: A Platform to Build Mobile Health Apps: The Personal Health Intervention Toolkit (PHIT)

Author(s): Eckhoff, RP (Eckhoff, Randall Peter); Kizakevich, PN (Kizakevich, Paul Nicholas); Bakalov, V (Bakalov, Vesselina); Zhang, YY (Zhang, Yuying); Bryant, SP (Bryant, Stephanie Patrice); Hobbs, MA (Hobbs, Maria Ann)

Source: JMIR MHEALTH AND UHEALTH **Volume:** 3 **Issue:** 2 **Article Number:** e46 **DOI:** 10.2196/mhealth.4202 **Published:** APR-JUN 2015

Language: English

Document Type: Article

Web of Science Categories: Health Care Sciences & Services; Medical Informatics

ISSN: 2291-5222

Record 53 of 64

Title: Utility of the T2 Mood Tracker Mobile Application Among Army Warrior Transition Unit Service Members

Author(s): Bush, NE (Bush, Nigel E.); Ouellette, G (Ouellette, Gary); Kinn, J (Kinn, Julie)

Source: MILITARY MEDICINE **Volume:** 179 **Issue:** 12 **Pages:** 1453-1457 **DOI:** 10.7205/MILMED-D-14-00271 **Published:** DEC 2014

Language: English

Document Type: Article
Web of Science Categories: Medicine, General & Internal
ISSN: 0026-4075
eISSN: 1930-613X

Record 54 of 64
Title: The Effectiveness of Mobile TeleHealth Biofeedback in Improving Symptoms of PTSD in Marines and Corpsmen Exposed to Combat
Author(s): Clemmons-James, DM (Clemmons-James, Dominiquie M.); Russoniello, CV (Russoniello, Carmen V.); Knauer, BB (Knauer, Brenda-Bart)
Source: APPLIED PSYCHOPHYSIOLOGY AND BIOFEEDBACK **Volume:** 39 **Issue:** 3-4 **Pages:** 312-312 **Published:** DEC 2014
Language: English
Document Type: Meeting Abstract
Web of Science Categories: Psychology, Clinical
ISSN: 1090-0586
eISSN: 1573-3270

Record 55 of 64
Title: Development of a Mobile Naturalistic Cognitive Assessment
Author(s): Evans, J (Evans, John); Russoniello, C (Russoniello, Carmen); Pougatchev, V (Pougatchev, Vadim); Bart-Knauer, B (Bart-Knauer, Brenda); Murray, N (Murray, Nick); Fish, M (Fish, Matt); Laing, B (Laing, Brenton); Clemmons-James, D (Clemmons-James, Dominiquie)
Source: APPLIED PSYCHOPHYSIOLOGY AND BIOFEEDBACK **Volume:** 39 **Issue:** 3-4 **Pages:** 313-313 **Published:** DEC 2014
Language: English
Document Type: Meeting Abstract
Web of Science Categories: Psychology, Clinical
ISSN: 1090-0586
eISSN: 1573-3270

Record 56 of 64
Title: Evaluating mobile apps for breathing training: The effectiveness of visualization
Author(s): Chittaro, L (Chittaro, Luca); Sioni, R (Sioni, Riccardo)
Source: COMPUTERS IN HUMAN BEHAVIOR **Volume:** 40 **Pages:** 56-63 **DOI:** 10.1016/j.chb.2014.07.049 **Published:** NOV 2014
Language: English
Document Type: Article
Web of Science Categories: Psychology, Multidisciplinary; Psychology, Experimental
ISSN: 0747-5632
eISSN: 1873-7692

Record 57 of 64

Title: The mobile emergency medical staff and the traumatic experience

Author(s): Laurent, A (Laurent, Alexandra)

Source: ANNALES MEDICO-PSYCHOLOGIQUES **Volume:** 172 **Issue:** 6 **Pages:** 457-462 **DOI:** 10.1016/j.amp.2012.03.015 **Published:** AUG 2014

Language: French

Document Type: Article

Web of Science Categories: Pharmacology & Pharmacy; Psychiatry; Psychology; Psychology, Multidisciplinary

ISSN: 0003-4487

eISSN: 1769-6631

Record 58 of 64

Title: Mobile Contingency Management as an Adjunctive Smoking Cessation Treatment for Smokers With Posttraumatic Stress Disorder

Author(s): Hertzberg, JS (Hertzberg, Jeffrey S.); Carpenter, VL (Carpenter, Vickie L.); Kirby, AC (Kirby, Angela C.); Calhoun, PS (Calhoun, Patrick S.); Moore, SD (Moore, Scott D.); Dennis, MF (Dennis, Michelle F.); Dennis, PA (Dennis, Paul A.); Dedert, EA (Dedert, Eric A.); Beckham, JC (Beckham, Jean C.)

Source: NICOTINE & TOBACCO RESEARCH **Volume:** 15 **Issue:** 11 **Pages:** 1934-1938 **DOI:** 10.1093/ntr/ntt060 **Published:** NOV 2013

Language: English

Document Type: Article

Web of Science Categories: Substance Abuse; Public, Environmental & Occupational Health

ISSN: 1462-2203

eISSN: 1469-994X

Record 59 of 64

Title: Virtual reality and mobile phones in the treatment of generalized anxiety disorders: a phase-2 clinical trial

Author(s): Repetto, C (Repetto, Claudia); Gaggioli, A (Gaggioli, Andrea); Pallavicini, F (Pallavicini, Federica); Cipresso, P (Cipresso, Pietro); Raspelli, S (Raspelli, Simona); Riva, G (Riva, Giuseppe)

Source: PERSONAL AND UBIQUITOUS COMPUTING **Volume:** 17 **Issue:** 2 **Special Issue:** SI **Pages:** 253-260 **DOI:** 10.1007/s00779-011-0467-0 **Published:** FEB 2013

Language: English

Document Type: Article

Web of Science Categories: Computer Science, Information Systems; Telecommunications

ISSN: 1617-4909

Record 60 of 64

Title: Enhancing behavioral health treatment and crisis management through mobile ecological momentary assessment and SMS messaging

Author(s): Smith, B (Smith, Brad); Harms, WD (Harms, William D.); Burres, S (Burres, Stephanie); Korda, H (Korda, Holly); Rosen, H (Rosen, Howard); Davis, J (Davis, Jamie)
Source: HEALTH INFORMATICS JOURNAL **Volume:** 18 **Issue:** 4 **Pages:** 294-308 **DOI:** 10.1177/1460458212445349 **Published:** DEC 2012
Language: English
Document Type: Article
Web of Science Categories: Health Care Sciences & Services; Medical Informatics
ISSN: 1460-4582
eISSN: 1741-2811

Record 61 of 64
Title: Mobile App Available for Supplemental PTSD Therapy
Author(s): [Anonymous] ([Anonymous])
Source: JOURNAL OF PSYCHOSOCIAL NURSING AND MENTAL HEALTH SERVICES **Volume:** 50 **Issue:** 10 **Pages:** 7-37 **Published:** OCT 2012
Language: English
Document Type: News Item
Web of Science Categories: Nursing
ISSN: 0279-3695

Record 62 of 64
Title: Mobile innovations, executive functions, and educational developments in conflict zones: a case study from Palestine
Author(s): Buckner, E (Buckner, Elizabeth); Kim, P (Kim, Paul)
Source: ETR&D-EDUCATIONAL TECHNOLOGY RESEARCH AND DEVELOPMENT **Volume:** 60 **Issue:** 1 **Pages:** 175-192 **DOI:** 10.1007/s11423-011-9221-6 **Published:** FEB 2012
Language: English
Document Type: Article
Web of Science Categories: Education & Educational Research
ISSN: 1042-1629
eISSN: 1556-6501

Record 63 of 64
Title: Mobile PTSD Care
Author(s): [Anonymous] ([Anonymous])
Source: JAMA-JOURNAL OF THE AMERICAN MEDICAL ASSOCIATION **Volume:** 306 **Issue:** 8 **Pages:** 815-815 **DOI:** 10.1001/jama.2011.1198 **Published:** AUG 24 2011
Language: English
Document Type: News Item
Web of Science Categories: Medicine, General & Internal

ISSN: 0098-7484
eISSN: 1538-3598

Record 64 of 64
Title: IDENTIFYING POSTTRAUMATIC STRESS DISORDER IN A PEDIATRIC MOBILE CLINIC: TWO CASE STUDIES
Author(s): Arnberger, R (Arnberger, R.); Vlahovich, K (Vlahovich, K.); Olteanu, A (Olteanu, A.)
Source: JOURNAL OF INVESTIGATIVE MEDICINE **Meeting Abstract:** 529 **Volume:** 58 **Issue:** 2 **Pages:** 506-506 **Published:** FEB 2010
Language: English
Document Type: Meeting Abstract
Conference Title: Southern Regional Meeting 2010
Conference Date: FEB 25-27, 2010
Conference Location: New Orleans, LA
Web of Science Categories: Medicine, General & Internal; Medicine, Research & Experimental
ISSN: 1081-5589
